# Relationship of Oral Health Literacy with Dental Caries and Oral Health Behavior of Children and Their Parents

**Published:** 2018-09

**Authors:** Reza Yazdani, Ehsan Nasr Esfahani, Mohammad Javad Kharazifard

**Affiliations:** 1 Associate Professor, Department of Community Oral Health, Research Center for Caries Prevention, School of Dentistry, Tehran University of Medical Sciences, International Campus, Tehran, Iran; 2 Dentist, Tehran, Iran; 3 PhD Candidate of Epidemiology, Dental Research Center, Dentistry Research Institute, Department of Epidemiology and Biostatistics, School of Public Health, Tehran University of Medical Sciences, Tehran, Iran

**Keywords:** Oral Health Literacy, Decayed Missing Filled Index, Parents, Dental Care for Children

## Abstract

**Objectives:**

This study sought to assess the relationship of oral health literacy (OHL) of parents with the decayed, missing, and filled teeth (DMFT) indices of themselves and their children.

**Materials and Methods:**

This cross-sectional study was performed on 258 children presenting with their parents to the Pediatric Dentistry and Orthodontics Departments of School of Dentistry, Tehran University of Medical Sciences in 2016. The parents were asked to fill out questionnaires related to OHL, oral health behaviors, and background information. Both parents and children were clinically examined to determine their DMFT indices according to the World Health Organization (WHO) criteria. A backward linear regression model was applied to assess the effect of demographic factors on OHL, behavioral habits, and DMFT. The Pearson’s bivariate correlation was used to assess the relationship of OHL, behavioral habits, and DMFT.

**Results:**

A significant linear correlation was noted between the OHL of the parents and the number of filled teeth in children (P=0.01). Only 48.5% of the parents had adequate OHL. Children whose parents had adequate OHL had a significantly higher number of fillings (P=0.03) and fewer missing teeth (P=0.04). Children whose parents had inadequate or marginal OHL had a significantly lower number of fillings (P=0.01) and more missing teeth (P=0.03).

**Conclusions:**

Higher OHL of parents seems to be significantly related to the mean DMFT of themselves and their children and enhances their oral health behavior. Programs must be implemented in developing countries, including Iran, to promote the OHL of parents and consequently improve the oral health status of children.

## INTRODUCTION

Oral health plays an important role in general health, and oral hygiene maintenance prevents many diseases [1]. More than half a century has passed since the identification of factors causing caries; however, dental caries is still a burden on the healthcare system of developing countries [2]. Oral health literacy (OHL) is a critical factor affecting the oral health of a community [3]. “The American Dental Association (ADA) defines OHL as the degree to which individuals have the capacity to obtain, process, and understand basic health information and services needed to make appropriate oral health decisions” [4].

The role of parents and particularly mothers in establishing and changing the health behavior of their children has been well documented [5]. Parents have the greatest effect on all aspects of health including physical and psychosocial health, and this effect starts at birth [6,7]. Inadequate OHL of parents is associated with children having high rates of dental caries and few dental fillings [8].

Sistani et al [8] in 2010 in Iran and Ramandeep et al [9] in 2013 in India evaluated the OHL of adults and showed that it was below the adequate level.

Haridas et al [10] in 2011 in India and Haerian Ardakani et al [11] in 2012 in Iran indicated a significant association among OHL, oral health status, and improvement of the decayed, missing, and filled teeth (DMFT) index. Vann et al [12] reported that the OHL of caregivers has a multidimensional impact on oral health outcomes in young children. In 2015, Khodadadi et al [7] in Iran evaluated the relationship of the OHL of parents and the oral health status of their children and showed that children of parents with a higher level of OHL had a better oral health status. In 2012, Brega et al [13] reported the same results in Navaho. In our country, Iran, less attention has been paid to prevention, while more emphasis has been placed by dentists on dental treatments [14].

Studies on OHL and its effects on oral health are still preliminary, and there is an obvious need for further research in this field [15]. Considering the significance of OHL and the role of parents in oral health behavior of children, this study aimed to assess the relationship of the OHL of parents with the DMFT indices of themselves and their children.

## MATERIALS AND METHODS

This descriptive, analytical cross-sectional study was conducted on 258 children (*n=*(*Z*1–*α*2)/2×p(1–*p*)/d2) presenting with their parents to the Pediatric Dentistry and Orthodontics Departments of School of Dentistry, Tehran University of Medical Sciences, Faculty of Dentistry, International Campus, Tehran, Iran, in 2016. The OHL questionnaire was filled out by the mothers voluntarily after signing an informed consent form. The validity and the reliability of this questionnaire have been previously confirmed in the Iranian and non-Iranian populations [8,16–18].

The children and their parents underwent dental clinical examinations according to the World Health Organization (WHO) standard diagnostic criteria [19] to determine the DMFT index by a senior dental student after calibration by a community oral health expert. The questionnaire included five sections. The first section included questions regarding general knowledge about oral and dental health. The second part included questions for the assessment of the ability to perceive dental instructions. The third section included questions regarding the assessment of personal decisions when encountering oral and dental problems as well as the perception of some expressions in the dental examination form. The fourth section included questions for the assessment of oral health behaviors, while the fifth section included questions regarding age, gender, level of education, number of family members, and occupational status. In the OHL questionnaire, there were a total of 17 correct choices. The OHL of those who acquired a score of 0–9 was considered inadequate, while scores 10–11 indicated moderate (marginal) OHL, and scores 12–17 indicated adequate OHL. The present study has been approved by the Research Ethics Committee of Tehran University of Medical Sciences (IR.TUMS.VCR.REC.1395.586). To assess the effect of demographic factors (age, gender, level of education, and number of family members), as independent variables, on OHL, behavioral habits (tooth brushing, dental visits, use of sugary snacks, and cigarette smoking) and DMFT, as dependent variables, a backward linear regression model was used for children and their mothers. The Pearson’s correlation bivariate was used to analyze the correlation of OHL variables, behavioral habits, and health indices. The level of significance was set at P<0.05. The statistical analyses were performed using SPSS 22 software program (IBM Co., Chicago, IL, USA).

## RESULTS

The mean age of the parents was 35.47 years (ranging from 20 to 60 years), and the mean age of the children was 8.39 years (ranging from 5 to 15 years). 14% of the children presented with their fathers, and 86% presented with their mothers. The frequency of adequate, inadequate, and marginal levels of the OHL of the parents was 48.5%, 28.3%, and 23.2%, respectively. A higher level of education of the parents was significantly correlated with an improvement in their OHL (B=2.208; Table 1)

**Table 1.** Linear correlation coefficient of oral health literacy (OHL) with the decayed, missing, and filled teeth (DMFT) index and its components in parents and children (n=258; Bivariate Pearson correlation)

** DMFT and its components **	** OHL **	** Adequate OHL **	** Marginal OHL **	** Inadequate OHL **
** DMFT. **	−0.002	−0.026	0.047	−0.015
** D.Parents **	−0.046	−0.055	−0.019	0.079
** M. Parents **	−0.082	−0.065	0.051	0.024
** F. Parents **	0.108	0.070	0.021	−0.097
** DMFT. Children **	−0.004	−0.035	0.060	−0.017
** D. Children **	−0.050	−0.051	0.035	0.024
** M. Children **	0.001	−0.060	0.113	−0.039
** F. Children **	0.070	0.015	0.055	−0.068
** dmft. Children **	−0.014	−0.007	0.005	0.004
** d. Children **	−0.053	−0.033	−0.015	0.050
** m. Children **	−0.057	−0.125 ^*^	0.131 ^*^	0.005
** f. Children **	0.152 ^*^	0.130 ^*^	0.016	−0.160 ^*^

* Significant

The mean DMFT was 9.75±4.36 for the parents, 6.33±3.80 for the primary teeth of the children, and 1.48±1.90 for the permanent teeth of the children. A higher level of education of the parents was significantly correlated with an increase in the number of filled teeth (F) of the parents (B=0.927), an increase in the number of filled primary teeth (f) in the children (B=0.295), a decrease in the number of missing teeth (M) in the parents (B=−0.975), and a decrease in the number of the primary teeth with caries (d) in the children (B=−0.871, P<0.05). The OHL of the parents had a significant correlation with the number of “f” in the children (P=0.014). Adequate OHL of the parents had a significant linear correlation with the number of “f” in the children (P=0.037) and an inverse correlation with missing teeth (m) in the children (P=0.045). Marginal OHL of the parents had a significant correlation with the “m” component in their children (P=0.036). Inadequate OHL of the parents had an inverse correlation with the “f” component in their children (P=0.010; Table 1). Only 48.5% of the parents had adequate OHL. Higher age of parents had a significant correlation with an increase in the DMFT of the parents (B=0.145) and children (B=0.079) and a reduction in the dmft of the children (B=−0.120). Also, the “M” component in male parents was significantly higher than that in female parents (B=−2.130, P<0.05; Table 2).

**Table 2.** Linear regression of the demographic factors with the oral health literacy (OHL), oral health behavior, and decayed, missing, and filled teeth (DMFT) indices of children and parents (n=258).

	** Age of parents (P-value) **	** Gender of parents (P-value) **	** Education of parents (P-value) **	** Number of family members (P-value) **
** OHL **	0.143	0.441	<0.001 ^**^	0.524
** DMFT. Parents **	0.001 ^**^	−0.089	−0.297	0.108
** D. Parents **	−0.435	−0.772	−0.129	0.339
** M. Parents **	0.254	0.001 ^*^	0.003 ^*^	0.414
** F. Parents **	0.002 ^**^	0.545	0.010 ^**^	0.587
** DMFT. Children **	<0.001 ^**^	0.658	0.531	0.096
** D. Children **	0.004 ^**^	0.434	0.987	0.293
** M. Children **	0.450	0.694	0.806	0.938
** F. Children **	0.008 ^**^	0.504	0.079	0.215
** dmft. Children **	0.002 ^*^	0.589	0.151	0.393
** d. Children **	<0.001 ^*^	0.551	0.018 ^*^	0.664
** m. Children **	0.272	0.667	0.843	0.157
** f. Children **	0.287	0.924	0.008 ^**^	0.756
** Oral health behaviors **	0.218	0.429	0.018 ^**^	0.943
** Use of toothbrush **	0.840	0.423	0.127	0.836
** Use of toothpaste **	0.166	0.009 ^*^	0.020 ^**^	0.279
** Last dental visit **	0.233	0.643	0.319	0.635
** No sugary snacks **	0.643	0.025 ^**^	0.977	0.216
** No smoking **	0.211	0.071	0.059	0.143

** Positive P&B P<0.05,

* Negative P&B P<0.05, Backward test linear regression

A significant correlation was noted between the number of decayed teeth (D) of the parents with the “D” component in their children (P=0.050). A significant inverse correlation existed between the “F” component in the parents and the “d” component in their children (P=0.032; Table 3).

**Table 3.** Correlation of decayed, missing, and filled teeth (DMFT) components in parents with DMFT components in children (n=258; Bivariate Pearson correlation).

** DMFT components **	** D. Parents **	** M. Parents **	** F. Parents **
** D. Children **	0.122 ^*^	0.034	−0.008
** M. Children **	0.024	−0.008	−0.040
** F. Children **	−0.069	−0.038	0.013
** d. Children **	<0.001	0.087	−0.133 ^*^
** m. Children **	−0.005	−0.089	0.031
** f. Children **	−0.037	0.085	0.105

* Significant

The OHL of the parents had a significant correlation with use of toothpaste (P=0.001), regular dental visits (P=0.001), and being a non-smoker (P=0.017). Inadequate OHL had an inverse correlation with oral health habits (P=0.005), use of toothpaste (P=0.001), the last dental visit (P=0.001), and being a non-smoker (P=0.038).

The time of the last dental visit of the parents was significantly correlated with the DMFT (P=0.005), with the “F” component (P<0.001), and with the “f” component in the children (P=0.044), while it had an inverse correlation with the “D” component in the parents (P=0.001). Lack of consumption of sugary snacks (P=0.028) and a non-smoking status (P=0.016) had a significant association with the “F” component in the parents. The non-smoking status had a significant inverse correlation with the “D” component in the parents (P=0.033; Table 4).

**Table 4.** Correlation of oral health behaviors of parents with the oral health literacy (OHL) and decayed, missing, and filled teeth (DMFT) indices of parents and their children (n=258; Bivariate Pearson correlation)

	** Oral health behaviors **	** Use of toothbrush **	** Use of toothpaste **	** Last dental visit **	** No sugary snacks **	** No smoking **
** OHL **	0.212 ^*^	0.039	0.201 ^*^	0.214 ^*^	−0.017	0.149 ^*^
** Adequate OHL **	0.103	0.020	0.085	0.094	−0.003	0.093
** Marginal OHL **	0.065	−0.042	0.123 ^*^	0.102	−0.027	0.028
** Inadequate OHL **	−0.175 ^*^	0.018	−0.210 ^*^	−0.200 ^*^	0.028	−0.129 ^*^
** DMFT. Parents **	0.076	0.090	−0.102	0.173 ^*^	0.022	0.004
** D. Parents **	−0.217 ^*^	−0.013	−0.076	−0.202 ^*^	−0.103	−0.133 ^*^
** M. Parents **	−0.047	−0.011	−0.110	0.057	−0.039	−0.053
** F. Parents **	0.290 ^*^	0.127 ^*^	0.036	0.297 ^*^	0.137 ^*^	0.150 ^*^
** DMFT. Children **	0.045	0.096	0.014	0.053	0.015	−0.039
** D. Children **	−0.039	0.032	−0.019	−0.013	0.009	−0.101
** M. Children **	0.021	0.005	0.021	−0.029	0.041	0.014
** F. Children **	0.147 ^*^	0.135 ^*^	0.056	0.125 ^*^	0.012	0.081
** dmft. Children **	−0.021	−0.072	−0.028	−0.027	0.037	0.002
** d. Children **	−0.017	−0.072	−0.016	−0.063	0.069	0.001
** m. Children **	−0.035	−0.046	−0.089	0.073	−0.067	0.043
** f. Children **	0.001	0.015	−0.005	0.093	−0.081	−0.012

* Significant

## DISCUSSION

This study aimed to find the relationship between the OHL of parents and the DMFT indices of themselves and their children presenting to the Pediatric Dentistry and Orthodontics Departments of School of Dentistry, Tehran University of Medical Sciences, Tehran, Iran, in 2016. The results showed that a higher OHL of parents was significantly correlated with a lower DMFT index in their children. Also, a higher OHL of parents resulted in a significant improvement in oral health behavior of themselves.

The present study was carried out among parents and their children presenting from a non-affluent area to the school of dentistry as clients.

In this study, OHL, as one of the important determinants of oral health, has been evaluated simultaneously with oral health behavior of parents and their children's oral health status, which is a positive feature of this study. However, this research has been conducted in one of the disadvantaged parts of the city; for this reason, there is no generalizability to the entire city. The results showed that a higher OHL of parents significantly related to an increase in the “f” component in their children. Also, children of parents with adequate OHL had higher “f” and lower "m" components. Children of parents with marginal and inadequate OHL had higher "m" and lower “f” components.

These results were in line with those reported by Khodadadi et al [7] who showed that children of parents with adequate OHL had a significantly higher number of filled teeth. The same findings were reported by Firmino et al [18], Miller et al [20], and Divaris et al [21]. Evidence shows that the OHL of parents is correlated with the oral health status of their children. In the current study, high OHL of the parents improved the dmft in their children.

According to the current results, 48.5% of the parents had adequate OHL, while others had inadequate or marginal OHL. These results were in agreement with those found by Sistani et al [8], Jones et al [22], and Kawamura et al [23]. These findings highlight the inadequate OHL of the general population of Iran and other countries. Since parents were the target group of our study and they play an important role in family health, inadequate OHL of parents is an important issue that must be necessarily addressed in strategic planning for educational programs to promote the OHL of the public.

Our results also revealed that a high level of education of parents significantly improved their OHL and oral health behavior. Also, parents with a higher level of education had significantly higher “F” and lower “M” components, while their children had significantly lower “d” and higher “f” components [24–26]. Sistani et al [8] also assessed the correlation between the level of education and OHL and reported the same results as ours. Moreover, studies on the correlation between the level of education and oral health behavior, including the study by Song et al [26], reported the same findings. Nematollahi et al [27] assessed the correlation between the level of education and the oral health status and reported results in accord with ours.

Evidence shows that the level of education directly correlates with OHL, and educated parents play an efficient role in the improvement of health behaviors of themselves and their family members. Our results, similar to those of previous studies, revealed a significant association between the level of education of parents and the oral health status of themselves and their children. These results indicate that parents with a higher level of education do whatever it takes for the prevention and treatment of oral and dental conditions of themselves and their children. According to the current results, parents with higher OHL had better oral health behaviors. Parents with inadequate OHL had a poor behavior in terms of using toothpaste, time of the last dental visit, and smoking status. Naghibi Sistani et al [28] and Ueno et al [29] reported the same results. Moreover, children of parents with better oral health behaviors had significantly lower “D” and higher “F” components, while the parents themselves had a significantly higher “F” component. The results of the study by Ueno et al [29] were in line with our findings.

Most parents participating in this study (62%) reported smoking once a day. Parents who brushed their teeth more frequently had a higher “F” component and their children had a higher “F” as well. The results reported by Bozorgmehr et al [30] and Ueno et al [29] were in agreement with ours. Also, 58% of the parents had visited a dentist during the past year, and the results showed that those with regular dental visits had a significantly higher OHL. This finding confirmed the results found by Al-Ansari et al [31]. Moreover, parents with regular dental visits had significantly lower “D” and higher “F” components. Their children had a significantly higher “F” as well. The results of the study by Ueno et al [29] were in agreement with our findings. Our results showed that parents with a higher OHL smoked significantly less than others, which was in accordance with the findings of the study by Holtzman et al [32]. Also, parents who smoked less had significantly lower “D” and higher “F” components. The same results were reported by de Araújo Nobre and Maló [33]. The results of studies conducted in Iran and other countries indicate that by an improvement in OHL, oral health behavior can be effectively changed. This change in oral health behavior of parents can improve their oral health status and that of their children. The current results showed that children whose parents had a lower “D” component had a significantly lower “D” as well. Also, children of parents with a higher “F” component had a significantly lower “d” component; Shin and Park [34] reported the same findings.

Overall, the results show a significant association between the oral health status of parents and that of their children.

## CONCLUSION

The current results showed that an improvement in the OHL of parents seems to improve the DMFT indices and oral health behaviors of themselves and their children. Therefore, programs are required to enhance the OHL of parents to promote the oral health of their children in developing countries, including Iran.
